# Optimization Design and Mechanical Performances of Plant-Mix Hot Recycled Asphalt Using Response Surface Methodology

**DOI:** 10.3390/ma16175863

**Published:** 2023-08-27

**Authors:** Honglin Liu, Jinping Wang, Weiwei Lu, Naitian Zhang

**Affiliations:** National Engineering Research Center of Highway Maintenance Technology, Changsha University of Science & Technology, Changsha 410114, China; hlliu@huuc.edu.cn (H.L.); wangjp@stu.csust.edu.cn (J.W.); zntcs@stu.csust.edu.cn (N.Z.)

**Keywords:** recycled asphalt, materials design, mechanical performance, response surface methodology, regression model

## Abstract

This study aimed to explore the influence of material design parameters on the physical and mechanical properties of recycled asphalt. A Box–Behnken design was employed to determine the optimal preparation scheme for 17 groups of recycled asphalt. The effects of styreneic methyl copolymer (SMC) regenerant content, styrene–butadiene–styrene (SBS)-modified asphalt content, and shear temperature on the mechanical properties of recycled asphalt were analyzed using conventional and high/low-temperature rheological tests. The optimal processing parameters were determined by a response surface model based on multiple response indexes. The results revealed that the SBS-modified asphalt content had the most significant effect on the penetration of recycled asphalt. An increase in SMC regenerant content led to a gradual decrease in the rutting factor, while SBS-modified asphalt content had the opposite effect. The usage of SMC regenerant helped to reduce non-recoverable creep compliance by adjusting the proportion of viscoelastic–plastic components in recycled asphalt. Furthermore, the stiffness modulus results indicated that the addition of SMC regenerant improved the recovery performance of recycled asphalt at a low temperature. The recommended contents of SMC regenerant and SBS-modified asphalt are 7.88% and 150%, respectively, with a shear temperature of 157.7 °C.

## 1. Introduction

Due to intricate traffic conditions and heavy traffic loads, the performance of asphalt pavement gradually deteriorates, failing to meet the demands of traffic. Consequently, rehabilitation projects of asphalt pavement generate approximately 100 million tons of reclaimed asphalt pavement (RAP) per year [1]. To address this issue, the utilization of RAP has gained significant attention [2]. The recycling of RAP offers benefits such as reduced energy consumption, preserved landfill space, minimized air pollution, conservation of non-renewable resources, and decreased production costs [3]. Previous research has indicated that while a high RAP content in asphalt mixtures improves resistance to rutting, it may negatively affect workability and promote fatigue cracking [4,5,6]. Moreover, the mechanical properties of recycled asphalt can be enhanced by incorporating a specific amount of regenerant [7]. Therefore, it is worth noting that an optimal value for both RAP content and regenerant amount may exist, enabling the enhancement of the mechanical properties of the asphalt mixture [8].

The plant-mix hot recycled technology of RAP encompasses a multitude of advantages, including the reutilization of waste materials; the conservation of non-renewable resources, such as mineral aggregate and asphalt; and the fulfillment of the requirements for low carbon development. Moreover, its construction process is straightforward, leading to its increasing popularity [9,10]. Numerous scholars have delved into the hot recycled technology of RAP. For example, Ferreira et al. [11] examined the influence of RAP content and aging heterogeneity on the properties of asphalt mixtures. Chen et al. [12] discovered a continuous improvement in the high-temperature performance of a plant-mix hot recycled asphalt mixture as the aged asphalt content increased. Lee et al. [13] examined the self-healing ability of RAP and evaluated the influence of different regenerants with a three-point bending test. Wan et al. [14] found that the use of hot recycled asphalt mixtures for the upper surface layer could improve the ability to resist the permanent strain of the pavement. Lee et al. [15] scrutinized the absolute viscosity of aged asphalt in RAP and the nominal maximum aggregate size of recycled aggregate, studying their influence on RAP performance. Hettiarachchi et al. [16] extracted aged asphalt from RAP and investigated the factors affecting the blending degree of recycled asphalt mixtures using Fourier transform infrared spectroscopy. Blanc et al. [17] explored the use of biomaterials as regenerants to achieve secondary utilization of RAP. Zhang et al. [18] evaluated the performance of four recycled asphalt mixtures with RAP contents of 0%, 15%, 30%, and 50%. Zaumanis M et al. [19] determined that the performance of a recycled asphalt mixture with 100% RAP approached the requirements of high modulus designs, but fell short of fully satisfying the fatigue, modulus, and rutting requirements. Meroni et al. [20] conducted a comprehensive evaluation of four recycled asphalt mixtures produced and laid in Virginia. Several studies have concluded that RAP enhances the rutting resistance and tensile strength of recycled asphalt mixtures while reducing moisture sensitivity. However, it has been observed that RAP inclusions can have a negative influence on the fatigue resistance of recycled asphalt mixtures [21]. Consequently, recycled asphalt technology has become a topic of great interest among scholars [22]. Recently, researchers have focused on studying the aging of asphalt in RAP to promote its regeneration and restore its fundamental properties [23]. It has been observed that utilizing less aged asphalt from RAP in recycled asphalt results in a brittle and hard state. This characteristic can be fully considered to optimize the performance of recycled asphalt.

Response surface methodology (RSM) represents a mathematical and statistical approach employed in the design of experiments, the mathematical modeling of variables (both univariate and multivariate), the evaluation of independent variables, and the optimization of procedures [24]. This method has been successfully applied in several fields, including civil engineering, mechanical and materials engineering, biology, and earth science [25,26,27]. In recent years, RSM has been found to have applications in the design and optimization of asphalt materials. For example, Rafiq et al. [28] optimized the content of binders and additives in stone mastic asphalt mixtures using RSM on the basis of volumetric properties and Marshall tests. Bala et al. [29] developed a performance-based statistical model utilizing RSM to optimize the content of asphalt binder and fine aggregates in hot mix asphalt. Guo et al. [30] employed RSM to assess the effects of waste engine oil content and gradation on indirect tensile strength (ITS) under dry and saturated conditions. Vatanparast et al. [31] optimized the asphalt content of a warm mix asphalt (WMA) mixture with RSM, considering volume characteristics and strength as responses. Relying on RSM, Taherkhani and Noorian [32] investigated the influence of preparation parameters, including gradation and aggregate types, on the ITS of WMA mixtures. Additionally, Khairuddin et al. [33] examined the influences of asphalt and polyurethane content on penetration, softening, and viscosity values using RSM. Previous research has demonstrated the successful application of RSM in establishing relationships between the parameters and performance characterization of asphalt materials. Hence, it is indeed feasible to optimize the preparation process of recycled asphalt utilizing RSM.

This study aimed to investigate the effects of preparation process parameters on the mechanical properties of recycled asphalt through RSM. The experimental data obtained in this study were utilized to establish a comprehensive multivariate quadratic regression equation and construct a three-dimensional response surface. These tools facilitated the identification of optimal values for the styrene–butadiene–styrene (SBS)-modified asphalt content, the styreneic methyl copolymer (SMC) regenerate content, and the shear temperature. Subsequently, the recycled asphalt was prepared according to the optimal design parameters. Both its conventional performance and rheological properties at high and low temperatures were assessed. Finally, the test results were compared with the predicted values generated from the regression equation, thereby further validating its reliability.

## 2. Materials and Methods

### 2.1. Materials

#### 2.1.1. Base Asphalt

In this study, a SBS (I-D) modified asphalt was selected as the base asphalt in recycled asphalt. Its main performance indexes are shown in Table 1.

#### 2.1.2. Regenerant

To recycle the asphalt from RAP, the aged asphalt, the regenerant, and the original asphalt were mixed in appropriate proportions. The recycled asphalt after this process can achieve the desired viscosity and the optimal performance for its intended applications. The regenerant plays a crucial role in adjusting the viscosity of the aged asphalt, modifying its colloidal structure, and improving its rheological properties. In this study, the SMC-III modifier was chosen as the regenerant, and its key performance indicators are outlined in Table 2.

### 2.2. Preparation of Recycled Asphalt

#### 2.2.1. Aging Simulation of Asphalt Binders

Generally, the asphalt pavement has suffered the impact of various environmental factors including light, oxygen, and humidity for a long time before undergoing the recycled process. Consequently, the asphalt binder obtained from RAP is aging badly. To replicate the long-term aging of asphalt in a laboratory, several accelerated aging methods have been developed in the last few decades. In this study, an accelerated aging test was performed using a pressure aging vessel.

Firstly, the original asphalt was subjected to softening in a rotary thin film oven (Shanghai Changji Geological Instrument Co., Ltd., Shanghai, China). Subsequently, the softened asphalt was transferred into aging pans (Shanghai Changji Geological Instrument Co., Ltd., Shanghai, China), with each pan containing a sample size of 50.0 g ± 0.5 g. These pans were placed on a burn-in tray. Once the temperature reached the tested value, the pressure vessel (Prentex Alloy Fabricators Inc, Dallas, TX, USA) was swiftly opened and the burn-in tray was inserted into the vessel. The vessel was sealed to initiate the aging process. The experiment was conducted under a pressure of 2.1 MPa ± 0.1 MPa and an aging duration of 20 h ± 10 min at a temperature of 100 °C. Upon completion of the predetermined aging time, the pressure inside the aging container was released to equalize the internal and external pressures. The long-term aged asphalt was obtained by removing the asphalt from the aging tray.

In response to the challenge of accurately predicting the long-term aging of asphalt binders, numerous researchers have undertaken the laboratory testing procedures to prepare aged asphalt specimens, serving as a viable substitute for the field asphalt derived from RAP. Qian et al. [34] suggested that although current laboratory aging methods might not completely replicate field conditions, the variability in performance of aged asphalt obtained through laboratory tests was comparatively lower. Moreover, Chen et al. [12] discovered that the influence of the aging asphalt content on the properties of the base asphalt aligned with the influence of the RAP content on the performance of the recycled asphalt mixture. Building upon these findings, this study employed the laboratory-prepared long-term aged asphalt as a substitute for the aged asphalt within RAP materials.

#### 2.2.2. Preparation of Recycled Asphalt

In this study, the procedure involved heating aged asphalt and SBS-modified asphalt to a molten state at 165 °C using an oven (Shanghai Changji Geological Instrument Co., Ltd., Shanghai, China). The SMC regenerative agent was gradually introduced to the aged asphalt while stirring to ensure thorough blending. After the addition was complete, the mixture was further stirred with a glass rod for an additional 10 min to enhance compatibility. Subsequently, the SBS-modified asphalt was weighed and added to the recycled asphalt, and the components were mixed using the glass rod. Finally, the mixture underwent shearing for 30 min using a high-speed shear apparatus at test temperature and a rotation rate of approximately 4000 r/min. The content of the SMC regenerative agent content, the SBS-modified asphalt, and the shear temperature were determined by the following test design.

### 2.3. Test Methods

#### 2.3.1. Penetration Test

Penetration serves as an indicator of the hardness, consistency, shear resistance, and viscosity of asphalt under specific circumstances. The test was operated by adjusting the thermostatic water (Shanghai Changji Geological Instrument Co., Ltd., Shanghai, China) bath to 25 °C and maintaining its stability as per specifications. Then, the sample was placed in the water bath for 1.5 h. After five seconds, the vertical penetration depth was measured on the displacement meter (Shanghai Changji Geological Instrument Co., Ltd., Shanghai, China) using a standard needle of 100 g, with an accuracy of 0.1 mm. The reading of depth represents the penetration of asphalt at 25 °C.

#### 2.3.2. Softening Point Test

The softening point plays a significant role in evaluating the temperature sensitivity of asphalt in road engineering. To determine this crucial parameter, a systematic procedure was followed. Firstly, the asphalt sample was allowed to cool naturally at room temperature for 30 min. Next, a steel ball was delicately placed on the surface of the sample, which was then carefully introduced into the softening point tester. The apparatus was heated gradually in a water bath, with the rate of increase set at 5 °C per minute. During this process, the asphalt gradually softened, leading to the sagging of the steel ball. The softening point was identified as the temperature at which the steel ball made contact with the surface of the bottom plate. To ensure accuracy, the softening point was meticulously measured and recorded with an accuracy of 0.5 °C. 

#### 2.3.3. Temperature Sweep Test

To assess the high-temperature rheological properties of recycled asphalt, a temperature sweep analysis was conducted using a dynamic shear rheometer (Anton Paar, Shanghai, China). This analysis aimed to acquire essential parameters such as the shear modulus G* and the phase angle δ of the regenerated asphalt composite, as well as the rutting factor G*/sin δ. The rutting factor can be defined as the ratio of the complex shear modulus to the sine of the phase angle. In this study, the strain control mode was selected for loading, employing a loading speed of 10 rad/s ± 0.1 rad/s. The temperature range for the test was set from 46 °C to 82 °C, with the temperature increasing at a controlled rate of 2 °C/min.

#### 2.3.4. Multiple Stress Creep Recovery Test

The determination of the non-recoverable creep compliance (*J_nr_*) of asphalt involved conducting a multi-stress creep recovery test in the stress control mode. This test was performed in two stress stages. The first stage employed a controlled stress level of 0.1 kPa, while the second stage utilized a controlled stress level of 3.2 kPa. During the test, repetitive loading was applied over a total of 30 cycles. Among these cycles, the initial 10 cycles served as a conditioning phase for the specimen under a stress level of 0.1 kPa; however, no data recording took place during this phase. The subsequent 10 cycles were conducted to record data while maintaining a stress level of 3.2 kPa. Finally, the remaining 10 cycles were loaded, and data recording was carried out. The recorded data were then utilized to calculate the irrecoverable creep compliance and creep recovery rate. Both the data recording and calculation processes were facilitated by the data acquisition system. With regard to the loading process for each cycle, the asphalt was subjected to constant stress loading for a duration of 1 s, followed by a recovery phase at zero stress for nine seconds. During the constant stress loading phase, stress and strain measurements were recorded at intervals of 0.1 s, while during the zero stress recovery phase, recordings were made at intervals of at least 0.45 s.

#### 2.3.5. Bending Creep Stiffness Test of Asphalt

To assess the toughness and resistance to cracking of a recycled asphalt binder at low temperature, a flexural creep stiffness test was conducted on the asphalt. The low temperature performance of the recycled asphalt was evaluated using the stiffness modulus (*S*) and creep rate (*m*) at a temperature of 60 °C. The stiffness modulus of the asphalt was kept below 300 MPa, and the creep rate was greater than 0.3. These criteria indicated that the asphalt exhibited good performance in low temperature conditions, showcasing its ability to withstand cracking.

### 2.4. Experimental Design of Response Surface Methodology

The RSM technique has been utilized to optimize the process parameters, minimize the number of required experiments, and evaluate the relationships between various influencing factors [35,36,37]. Unlike traditional single-factor and orthogonal experiments, RSM establishes a multivariate quadratic regression equation that relates the influencing factors to the response values [35,36]. RSM offers two methods in terms of experimental designs, including the Box–Behnken and the central composite design. In this study, the Box–Behnken method was employed with the SBS-modified asphalt content, the SMC regenerant content, and the shear test temperature designated as independent variables, which were denoted as *X*_1_, *X*_2_, and *X*_3_, respectively. The range of SMC regenerant content was set at 4% to 12%. The range of SBS-modified asphalt content was 66% to 150%, while the range of shear temperature was 150 °C to 170 °C. The coded levels of the independent variables are presented in Table 3.

Based on the Box–Behnken design, 17 groups of preparation parameters of recycled asphalt were determined. The SMC regenerant content and SBS-modified asphalt content were determined as a percentage of the long-term aged asphalt by mass. The preparing scheme for recycled asphalt samples is outlined in Table 4. Following modeling with RSM, an analysis of variance (ANOVA) was conducted to quantitatively examine the influence of the preparation process parameters on the properties of recycled asphalt. The suitability of the model was assessed through residuals, F-values, and *p*-values obtained from the ANOVA.

## 3. Results

The evaluation of asphalt recycled technology mainly depends on the physical and mechanical properties of recycled asphalt. The performance test results of recycled asphalt with different material design parameters are presented in Table 5.

### 3.1. Characterization of Recycled Asphalt Performance

To investigate the differences between the regression equations using different methods, an ANOVA test was conducted on the same dataset. The null and alternative hypotheses for each characteristic are presented in Table 6, while the results of the ANOVA for each characteristic are shown in Table 7. The null hypotheses can be rejected since the computed *p*-value is less than 0.05, which suggests that the quadratic model adequately represents the penetration at a significance level of less than 0.05.

#### 3.1.1. Penetration Test Results and Analysis

Using the penetration test data, the least square method was utilized to establish a multiple quadratic regression equation, as shown in Equation (1). To assess the significance of each factor within the fitted equation, a variance test was executed to eliminate elements that are not significant. The null hypothesis assumes that the parameters exhibit significance, while the alternative hypothesis suggests the parameters are not significant. If the *p*-value is less than 0.05, the null hypothesis is embraced; otherwise, the alternative hypothesis is favored. As shown in Table 8, the ANOVA results reveal a high significance level for the model (*p* < 0.0001). The predicted values derived from the regression equation exhibited a strong correlation with the experimental values (R^2^ = 1.0000), thereby confirming the excellence of the model in accurately reflecting the actual outcomes. The lack of fit attributed to pure error is not significant.

(1) $$\begin{array}{l} Pen=61.40+10.72X_{1}-13.24X_{2}-1.70X_{3}-2.72X_{1}X_{2}\\ \;-3.15X_{1}X_{3}+7.40X_{2}X_{3}+2.84X_{1}^{2}+4.46X_{2}^{2}+9.39X_{3}^{2} \end{array}$$

where, *Pen* is 25 °C penetration (0.1 mm); *X*_1_ is the content of the SMC regenerant; *X*_2_ is the content of the SBS-modified asphalt; and *X*_3_ is the shear temperature.

The *p*-value represents the likelihood of an event occurring and is important in determining the significance of a sample. If the *p*-value exceeds 0.05, it implies that the observed difference may be attributed to factors beyond mere sampling errors, rendering the item not significant. Conversely, a *p*-value below 0.05 indicates statistical significance. In this specific scenario, the content of SMC regenerant, SBS-modified asphalt, and shear temperature significantly influence the penetration. Notably, the regression equation for penetration remains unaltered, as all the *p*-values hold a value of 0.0001.

#### 3.1.2. Softening Point Test Results and Analysis

The softening point test results were analyzed using the least square method to fit the multiple quadratic regression equation (as shown in Equation (2)), with *Spt* representing the softening point. An ANOVA was performed on the model, and the results are shown in Table 9. The null hypothesis states that the parameters are significant, while the alternative hypothesis suggests that the parameters are not significant. The null hypothesis is accepted when the *p*-value is less than 0.05. The statistical analysis demonstrated that the model holds immense significance (*p* < 0.0001). Specifically, the content of SMC regenerant, SBS-modified asphalt, and the shear temperature are identified as significantly influencing the softening point. The strong correlation between predicted and experimental values (R^2^ = 0.9860) confirm the accuracy of the model in reflecting the actual outcomes.

(2) $$\begin{array}{l} Spt=69.29+1.99X_{1}+3.14X_{2}-1.82X_{3}-0.4875X_{1}X_{2}\\ \;+2.21X_{1}X_{3}+3.33X_{2}X_{3}+2.04X_{1}^{2}+1.91X_{2}^{2}-3.84X_{3}^{2} \end{array}$$

Based on the results in Table 9, the fitting function of the softening point after eliminating the terms that are not significant can be reconstructed in Equation (3).

(3) $$\begin{array}{l} Spt=69.29+1.99X_{1}+3.14X_{2}-1.82X_{3}+2.21X_{1}X_{3}\\ \;+3.33X_{2}X_{3}+2.04X_{1}^{2}+1.91X_{2}^{2}-3.84X_{3}^{2} \end{array}$$

#### 3.1.3. Temperature Scanning Test Results and Analysis

Utilizing the test results in Table 5, the multiple quadratic regression equation was fitted using the least square method. This equation is denoted as Equation (4).

(4) $$\begin{array}{l} G^{*}/\sin\delta =2.37-0.3863X_{1}+0.3950X_{2}-0.1162X_{3}+0.0275X_{1}X_{2}\\ \;-0{.0050X}_{1}X_{3}+0{.0775X}_{2}X_{3}-0.099X_{1}^{2}+0.2035X_{2}^{2}-0.284X_{3}^{2} \end{array}$$

An ANOVA was performed on the model, and the results are shown in Table 10. The null hypothesis suggests that the parameters hold significance, while the alternative hypothesis posits their insignificance. If the *p*-value is less than 0.05, the null hypothesis is accepted; otherwise, the alternative hypothesis is accepted. Notably, as observed from Table 10, a *p*-value of less than 0.0001 for the model signifies its utmost significance. The constituents of SMC regenerant, SBS-modified asphalt, and the shear temperature notably influence the rutting factor. Moreover, the predicted value strongly correlated with the experimental value (R^2^ = 0.9857), effectively reflecting the actual outcomes.

The term, which is not significant, is removed, and the function of the rutting factor obtained anew is shown in Equation (5).

(5) $$G^{*}/\sin\delta =2.37-0.3863X_{1}+0.395X_{2}-0.1162X_{3}-0.099X_{1}^{2}+0.2035X_{2}^{2}-0.284X_{3}^{2}$$

#### 3.1.4. Multiple Stress Creep Recovery Test Results and Analysis

Using the least squares method, a multiple quadratic regression equation, depicted as Equation (6), was fitted for the multiple stress creep recovery tests.

(6) $$\begin{array}{l} J_{nr}=0.7300+0.0600X_{1}+0.0425X_{2}-0.0375X_{3}-0.1300X_{1}X_{2}\\ \;-0.0700X_{1}X_{3}+0.0600X_{2}X_{3}+0.0600X_{1}^{2}+0.0250X_{2}^{2}+0.0450X_{3}^{2} \end{array}$$

An ANOVA analysis was conducted on the model, and the results were detailed in Table 11. The null hypothesis posits the significance of the parameters, while the alternative hypothesis assumes their insignificance. Should the *p*-value fall below 0.05, the null hypothesis is accepted; otherwise, the alternative hypothesis is favored. Notably, the model yielded a *p*-value of less than 0.0001, indicating its significance. The content of SMC regenerant, SBS-modified asphalt, and the shear temperature were observed to wield significant influence over non-recoverable creep compliance. The predicted values strongly correlated with the experimental values (R^2^ = 0.9984), and the lack of fit, attributed to pure error, was insignificant. Hence, the model fit is deemed satisfactory to accurately reflect the real-world outcomes. Given that all the *p*-values are below 0.05, the regression equation for non-recoverable creep compliance remains unchanged.

#### 3.1.5. Test Results and Analysis of Flexural Creep Stiffness of Asphalt

Based on the flexural creep stiffness test results, a multiple quadratic regression equation was fitted using the least squares method, as presented in Equations (7) and (8).

(7) $$\begin{array}{l} m=0.3200+0.0662X_{1}+0.0438X_{2}-0.0225X_{3}-0.0275X_{1}X_{2}\\ \;+0.0050X_{1}X_{3}+0.0250X_{2}X_{3}+0.0363X_{1}^{2}-0.0137X_{2}^{2}-0.0113X_{3}^{2} \end{array}$$

(8) $$\begin{array}{l} S=53.40-18.33X_{1}-12.73X_{2}-4.80X_{3}+12.29X_{1}X_{2}\\ \;+2.330X_{1}X_{3}+3.68X_{2}X_{3}+2.01X_{1}^{2}+3.53X_{2}^{2}+16.09X_{3}^{2} \end{array}$$

where *m* is the creep rate and *S* is the stiffness modulus.

ANOVA analyses were performed on both the creep rate and stiffness modulus models, and the corresponding results are displayed in Table 12 and Table 13, respectively. The null hypothesis asserts the significance of the parameters, while the alternative hypothesis suggests their insignificance. Acceptance of the null hypothesis occurs when the *p*-value is below 0.05; otherwise, the alternative hypothesis is favored. The statistical analysis unveiled that the model exhibits remarkable significance, as evidenced by the *p*-value of less than 0.0001. Notably, the content of SMC regenerant, SBS-modified asphalt, and the shear temperature significantly influence the creep rate, while the content of SMC regenerant and SBS-modified asphalt exert a noteworthy influence on the stiffness modulus. Furthermore, a strong correlation between the predicted and experimental values is observed, with R^2^ values of 0.9944 and 0.9459 for the creep rate and stiffness modulus, respectively. The result signifies that the model accurately reflects the actual results with high precision.

Removing the non-significant terms, the functions of creep rate and stiffness modulus are presented in Equations (9) and (10), respectively.

(9) $$\begin{array}{l} m=0.3200+0.0662X_{1}+0.0438X_{2}-0.0225X_{3}-0.0275X_{1}X_{2}\\ \;+0.0250X_{2}X_{3}+0.0363X_{1}^{2}-0.0137X_{2}^{2}-0.0113X_{3}^{2} \end{array}$$

(10) $$S=53.4-18.33X_{1}-12.73X_{2}+12.29X_{1}X_{2}+16.09X_{3}^{2}$$

### 3.2. Response Surface Interaction Analysis

#### 3.2.1. Penetration

**Figure 1:**
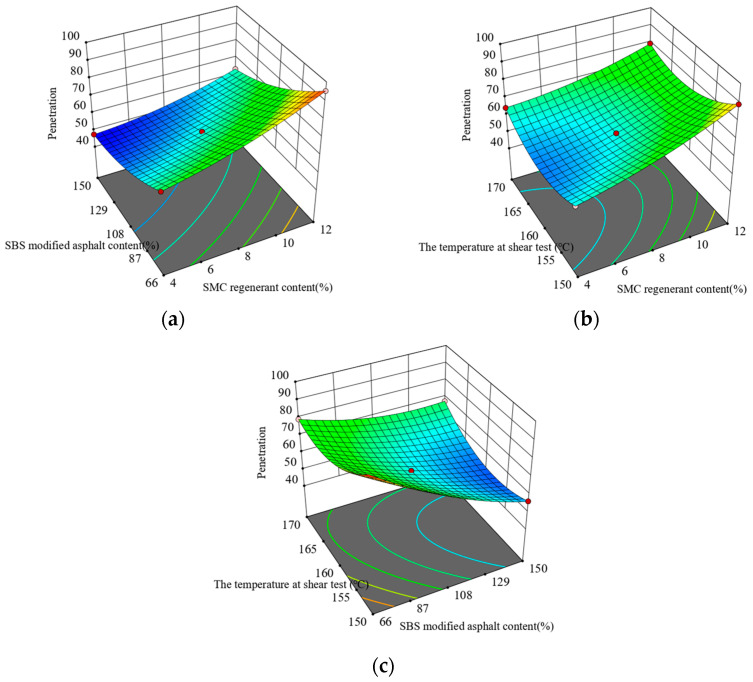
Interaction between test factors and penetration: (**a**) regenerate agent and original SBS-modified asphalt content; (**b**) regenerant content and the temperature at shear test; and (**c**) the temperature at shear test and SBS-modified asphalt content.

Figure 1 presents a response surface plot showcasing the outcomes of the penetration test. The results illustrate that with an increase in the temperature during the shear test, the permeability shows an initial rise followed by a subsequent decline. In contrast, the levels of SMC regenerant and SBS-modified asphalt remain constant throughout. Moreover, elevating the quantity of SBS-modified asphalt decreases penetration, while the addition of SMC regenerant produces the opposite effect.

#### 3.2.2. Softening Point

**Figure 2:**
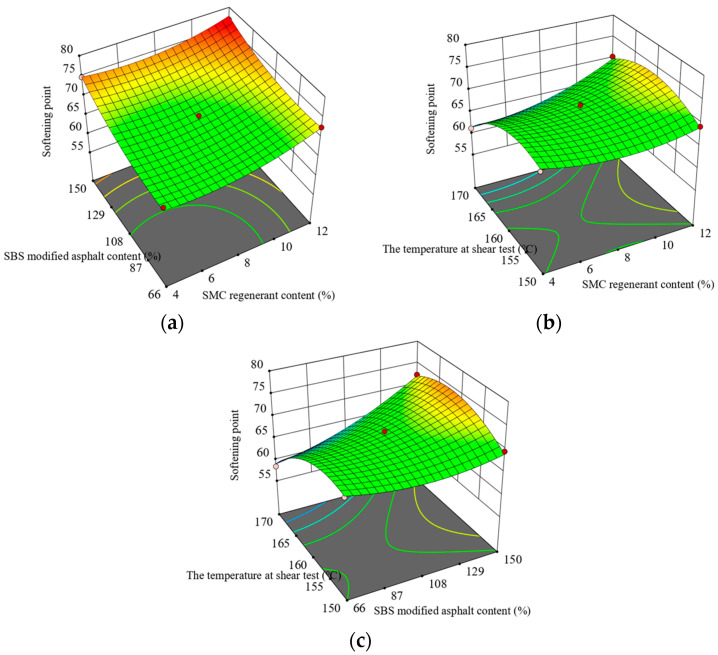
Interaction between experimental factors and softening point: (**a**) regenerate agent and original SBS-modified asphalt content; (**b**) regenerant content and the temperature at shear test; and (**c**) the temperature at shear test and SBS-modified asphalt content.

Figure 2 shows a response surface plot representing the outcomes of the softening point analysis, indicating that while SBS-modified asphalt is kept at a constant level, the softening point increases as the SMC regenerant content rises. Likewise, when the SMC regenerant content remains consistent, the softening point experiences an increase with higher quantities of SBS-modified asphalt. Specifically, when the content of SMC regenerant is fixed at a specific value and the content of SBS-modified asphalt is set at 108%, a decrease in the softening point is observed as the shearing temperature increases for SBS-modified asphalt contents below 108%. However, when the content of SBS-modified asphalt surpasses 108%, the softening point initially rises and then declines with an increase in shearing temperature.

#### 3.2.3. Temperature Sweep Test

**Figure 3:**
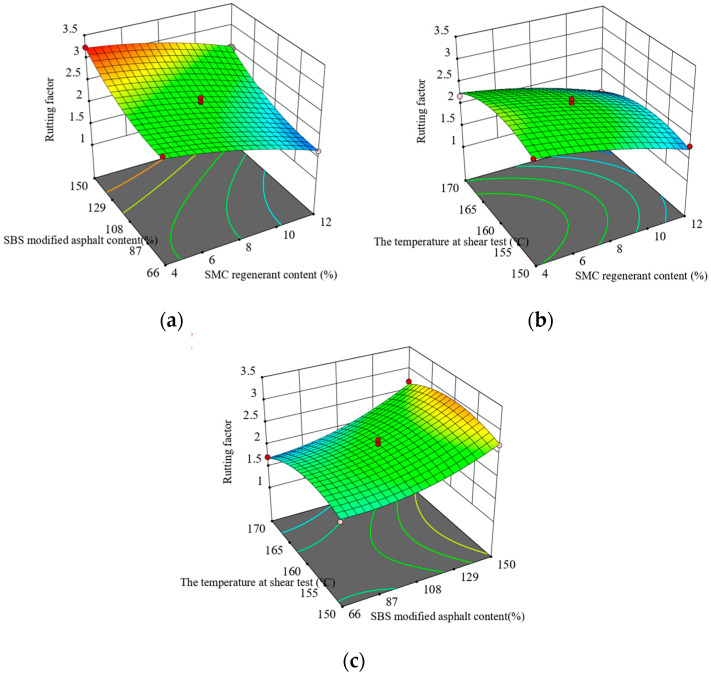
Interaction between test factors and rutting factor: (**a**) regenerate agent and original SBS-modified asphalt content; (**b**) regenerant content and the temperature at shear test; and (**c**) the temperature at shear test and SBS-modified asphalt content.

Figure 3 indicates a decrease in the rutting factor as the content of regenerant and SBS-modified asphalt increases. Additionally, the rutting factor shows a decrease with higher shear temperatures.

#### 3.2.4. Multiple Stress Creep Recovery Test

**Figure 4:**
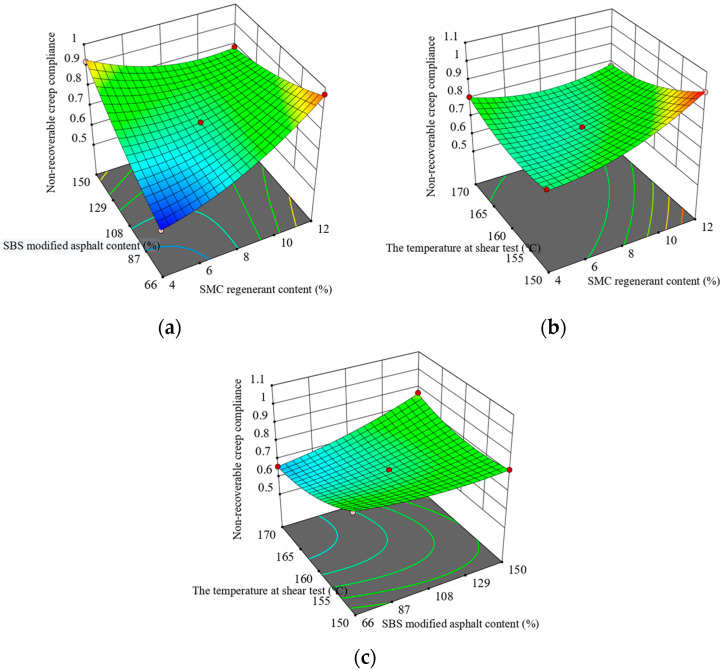
Interaction between test factors and non-recoverable creep compliance: (**a**) regenerate agent and original SBS-modified asphalt content; (**b**) regenerant content and the temperature at shear test; and (**c**) the temperature at shear test and SBS-modified asphalt content.

Based on the results of the multiple stress creep recovery test, Figure 4 illustrates a response surface plot. It is apparent that when the content of SBS-modified asphalt is lower than 111.41%, and the shear temperature remains constant, the non-recoverable creep compliance demonstrates an increase as the SMC regenerator content increases. However, when the content of SBS-modified asphalt exceeds 111.41%, the non-recoverable creep compliance shows decreases with an increase in the content of SMC regenerant. Additionally, as the shear temperature increases, and the content of SMC regenerant is high while the SBS-modified asphalt content is low, the irrecoverable creep compliance experiences a decrease.

#### 3.2.5. Multiple Stress Creep Recovery Test

**Figure 5:**
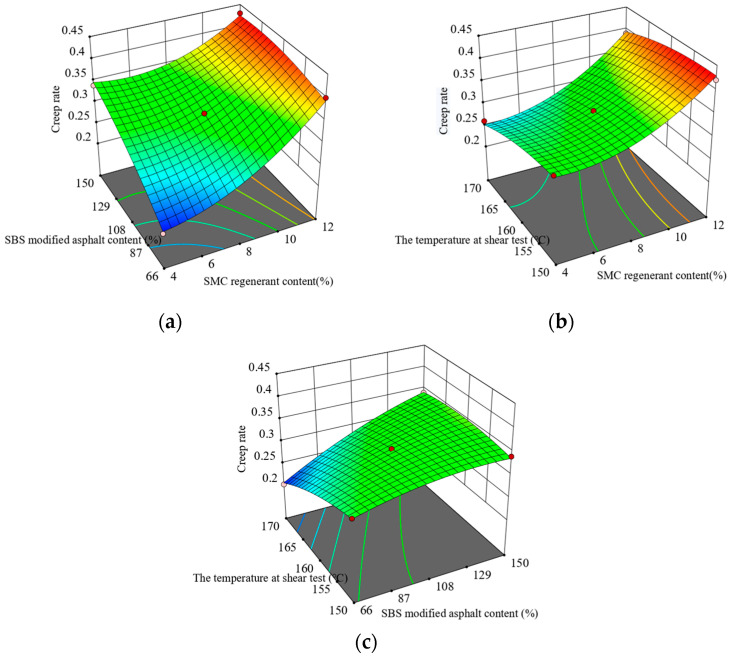
Interaction between test factors and creep rate of recycled asphalt: (**a**) regenerate agent and original SBS-modified asphalt content; (**b**) regenerant content and the temperature at shear test; and (**c**) the temperature at shear test and original SBS-modified asphalt content.

**Figure 6:**
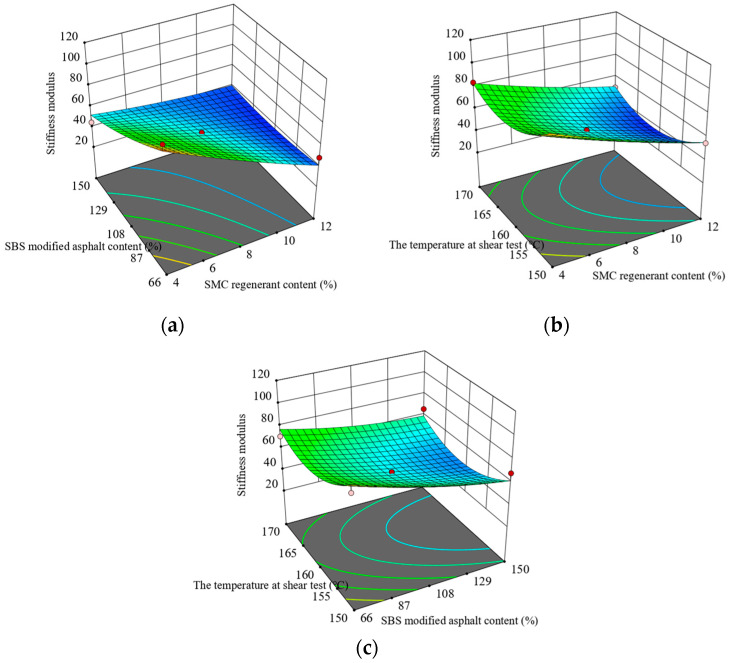
Interaction between test factors and stiffness modulus of recycled asphalt: (**a**) regenerate agent and original SBS-modified asphalt content; (**b**) regenerant content and the temperature at shear test; and (**c**) the temperature at shear test and original SBS-modified asphalt content.

Figure 5 and Figure 6 illustrate the response surface plots derived from the creep test results. The results reveal a gradual elevation in the creep rate of asphalt as the content of SMC regenerant increases, while maintaining a constant content of SBS-modified asphalt. Conversely, when the SMC regenerant content remains constant, the creep velocity initially decreases and then increases as the shear temperature rises. At a specific shear temperature and SMC regenerant content, the stiffness modulus of SBS-modified asphalt diminishes with an increase in the content of SBS-modified asphalt. Furthermore, when the shear temperature and SBS-modified asphalt content remain constant, the stiffness modulus decreases as the content of SMC regenerant increases. When the content of SMC regenerant and SBS-modified asphalt remains constant, the stiffness modulus encounters an initial decline followed by an increase with an elevated shear temperature.

### 3.3. Performance Optimization and Model Verification of Recycled Asphalt

Based on the above test analysis, this study has successfully determined the optimal design parameters of recycled asphalt. Specifically, the SMC regenerant content is 7.88% (8% in subsequent investigations), the content of SBS-modified asphalt has been increased to 150%, and the shear temperature has been set at 157.68 °C (160 °C in subsequent analysis). By employing these carefully chosen parameters, the projected values for key performance indicators of recycled asphalt under stress levels of 0.1 kPa and 3.2 kPa are as follows: 52.1 (0.1 mm) for penetration, 73.88 °C for softening point, 2.94 kPa for rutting factor, 0.347 for creep rate, 44.95 MPa for stiffness modulus, 0.796 kPa^−1^ for average non-recoverable creep compliance at 0.1 kPa, and 1.185 kPa^−1^ for average non-recoverable creep compliance at 3.2 kPa.

To validate the optimal preparation process parameters obtained through response surface methodology (RSM), recycled asphalt was prepared using the specified conditions: 8% SMC regenerant content, 150% SBS-modified asphalt, and a shear test temperature of 160 °C. Subsequently, several performance tests were conducted, including conventional performance evaluation and high and low-temperature rheological performance assessment. The outcomes of these tests are presented in Table 14. In the table, *m* denotes the creep rate and *S* denotes the stiffness modulus. The comparison between the predicted and actual values of the key recycled asphalt indexes, along with the maximum error of 5.04%, falls within an acceptable range. 

## 4. Conclusions

This study employed the response surface method to investigate the effects of SMC regenerant content, SBS-modified asphalt content, and shear temperature on the mechanical properties of recycled asphalt. The application of RSM in optimizing recycled asphalt design was demonstrated through constructing regression equations, significance testing, prediction of optimal mix proportions, and experimental verification. The main findings are as follows.

(1) When the content of SMC regenerant or SBS-modified asphalt is fixed, the penetration exhibits a trend of initially increasing and then decreasing with an increase in shear temperature. With the increase in SBS-modified asphalt content, the penetration decreases, and with the increase in shear temperature, the penetration first increases and then decreases. Among the three influencing factors, SBS-modified asphalt content has the greatest impact on penetration. The increasing content of SBS-modified asphalt and SMC regenerant decreased the softening point of recycled asphalt. The softening point tends to decrease with an increase in shear temperature.
(2) When the content of SMC regenerant and SBS-modified asphalt is fixed, an increase in shear temperature initially increases, and then the rutting factor decreases. The increasing content of SMC regenerant leads to a gradual decrease in the rutting factor, while SBS-modified asphalt content has the opposite effect. In a comprehensive comparison, SBS-modified asphalt content has the most significant effect on the rutting factor. With a constant content of SMC regenerant and SBS-modified asphalt, an increase in shear temperature results in a first decrease and then an increase in non-recoverable creep compliance. The addition of SMC regenerant reduces the non-recoverable creep compliance by regulating the proportion of viscoelastic and plastic components in recycled asphalt.
(3) The stiffness modulus of recycled asphalt gradually decreases with an increase in the content of SBS-modified asphalt, while keeping the shear temperature and SMC regenerant content constant. Similarly, with constant shear temperature and SMC regenerant content, the stiffness modulus gradually decreases with an increase in SMC regenerant content. Additionally, the creep rate increases gradually with a constant content of SMC regenerant. Notably, when the content of SBS-modified asphalt is 66%, the creep rate increases by 50% as the SMC regenerant content increases from 4% to 12%. These findings highlight the significant enhancement of low-temperature properties through the beneficial use of SMC regenerant.

In summary, this study contributes to the field of road engineering by demonstrating the feasibility of RSM in optimizing recycled asphalt design. The study findings emphasize the effects of the shear temperature, the SBS-modified asphalt content, and the SMC regenerant content on the performance of recycled asphalt, including penetration, softening point, rut factor, and stiffness modulus. These insights provide valuable guidance for the development of sustainable and high-performance asphalt pavements.

## Figures and Tables

**Table 1:** General physical properties of SBS (I-D) modified asphalt.

Indicators		Test Results	Specification Requirement	Test Method
Penetration at 25 °C, 100 g, and 5 s (0.1 mm)		71	60–80	ASTM D5
Penetration index		0.15	−1.5–1.0	ASTM D5
Ductility at 5 cm/min and 5 °C (cm)		>150	≥100	ASTM D113
Softening point (°C)		48	≥46	ASTM D36
Kinematic viscosity at 135 °C (Pa·s)		2.45	≤3	ASTM D2171
Solubility (%)		99.78	>99.5	ASTM D2042
Flash point (°C)		310	≥260	ASTM D92
Residue from the film oven test *	Mass loss (%)	−0.04	≤±1.0	ASTM D402
	Penetration at 25 °C (%)	78	≥65	ASTM D5
	Ductility at 5 °C (cm)	22	≥20	ASTM D113

* Notes: The film oven test was conducted by situating a vessel containing asphalt in a film heating oven. The test temperature was set at 163 °C, and the duration was 5 h. The asphalt film after the test was defined as the residue from the film oven test. This test aimed to determine the mass changes of asphalt films after heating. Additionally, the changes in the properties of asphalt residue, including penetration and ductility, were measured to assess the aging resistance performance of original asphalt.

**Table 2:** Basic properties of SMC regenerant.

Performance	Test Results
Appearance	Yellow-brown viscous liquid
Density (g/cm^3^)	0.88
Flash point by open cup method (°C)	70
hydrocarbon content of rub (%)	84
Viscosity at 25 °C (Pa·s)	0.71

**Table 3:** Factor and level coding table for Box–Behnken design.

Factor	Symbol	Unit	Code Level		
			−1	0	1
SMC regenerant content	*X*_1_	%	4	8	12
SBS-modified asphalt content	*X*_2_	%	66	108	150
Shearing temperature	*X*_3_	°C	150	160	170

**Table 4:** Preparation scheme of recycled asphalt samples for each group.

Serial Number	Recycled Content (%)	Ratio of SBS-Modified Asphalt (%)	The Temperature at Shear Test (°C)
1	8.00	66.00	150.00
2	12.00	150.00	160.00
3	8.00	150.00	170.00
4	8.00	108.00	160.00
5	12.00	108.00	170.00
6	8.00	108.00	160.00
7	8.00	108.00	160.00
8	4.00	66.00	160.00
9	8.00	108.00	160.00
10	12.00	108.00	150.00
11	8.00	66.00	170.00
12	8.00	108.00	160.00
13	8.00	150.00	150.00
14	4.00	108.00	170.00
15	4.00	108.00	150.00
16	4.00	150.00	160.00
17	12.00	66.00	160.00

**Table 5:** Performance test results of recycled asphalt.

Serial Number	Penetration (0.1 mm)	Softening Point (°C)	Rutting Factor (G*/sinδ)	Non-Recoverable Creep Compliance (kPa^−1^)	Creep Rate (%)	Stiffness Modulus (Pa)
1	97.63	69.1	2.03	0.85	0.3	87.65
2	63.41	77.1	2.46	0.79	0.43	34.46
3	67.67	72.3	2.7	0.87	0.34	65.75
4	61.4	69.1	2.47	0.73	0.32	53.4
5	79.58	70.4	1.47	0.78	0.39	49.8
6	61.4	70.3	2.39	0.73	0.32	53.4
7	61.4	69.1	2.35	0.73	0.32	53.4
8	68.53	68.5	2.54	0.58	0.2	108
9	61.4	69.1	2.26	0.73	0.32	53.4
10	89.2	69.3	1.79	1	0.42	54.9
11	79.35	58.5	1.72	0.66	0.2	70.8
12	61.4	69.1	2.37	0.73	0.32	53.4
13	56.36	69.6	2.7	0.82	0.34	67.9
14	64.36	61.3	2.19	0.81	0.26	83.5
15	61.38	69	2.49	0.75	0.31	97.8
16	47.5	74.8	3.24	0.92	0.34	44.9
17	95.33	72.7	1.65	0.97	0.4	48.4

**Table 6:** Summary of the null and alternative hypotheses.

Asphalt Performance Index	Test Hypotheses	
	Null	Alternative
Penetration	Quadratic model has no influence on asphalt performance index.	Quadratic model has influence on asphalt performance index.
Softening point		
Rutting factor		
Non-recoverable creep compliance		
Creep rate		
Stiffness modulus		

**Table 7:** Summary of the one-way ANOVA results.

Asphalt Performance Index	Null Hypothesis	
	F	*p*-Value
Penetration	45,444.21	<0.0001
Softening point	50.95	<0.0001
Rutting factor	27.65	0.0003
Non-recoverable creep compliance	225.37	<0.0001
Creep rate	40.75	<0.0001
Stiffness modulus	8.16	0.0110

**Table 8:** ANOVA of regression model of penetration.

Variance Source	Sum of Squares	Degree of Freedom	Mean Square	F Value	*p*-Value	Significance
Model	3162.58	9	351.40	90,350.25	<0.0001	significant
Residual error	0.0272	7	0.0039	−	−	−
Total	3162.61	16	−	−	−	−
*X*_1_	919.13	1	919.13	2.363 × 10^5^	<0.0001	significant
*X*_2_	1401.85	1	1401.85	3.604 × 10^5^	<0.0001	significant
*X*_3_	23.15	1	23.15	5953.28	<0.0001	significant
*X*_1_ *X*_2_	29.65	1	29.65	7623.00	<0.0001	significant
*X*_1_ *X*_3_	39.69	1	39.69	10,204.96	<0.0001	significant
*X*_2_ *X*_3_	218.89	1	218.89	56,280.78	<0.0001	significant
*X* ^2^_1_	33.84	1	33.84	8701.07	<0.0001	significant
*X* ^2^_2_	83.66	1	83.66	21,510.44	<0.0001	significant
*X* ^2^_3_	371.65	1	371.65	95,556.47	<0.0001	significant
Adjust R^2^ = 1.0000						

**Table 9:** Variance analysis of softening point regression model.

Variance Source	Sum of Squares	Degree of Freedom	Mean Square	F Value	*p*-Value	Significance
Model	292.23	9	32.47	54.93	<0.0001	significant
Residual error	4.14	7	0.5911	−	−	−
Total	296.36	16	−	−	−	−
*X*_1_	31.60	1	31.60	53.46	0.0002	significant
*X*_2_	79.07	1	79.07	133.76	<0.0001	significant
*X*_3_	26.46	1	26.46	44.77	0.0003	significant
*X*_1_ *X*_2_	0.9506	1	0.9506	1.61	0.2453	not significant
*X*_1_ *X*_3_	19.58	1	19.58	33.13	0.0007	significant
*X*_2_ *X*_3_	44.22	1	44.22	74.82	<0.0001	significant
*X* ^2^_1_	17.57	1	17.57	29.72	0.0010	significant
*X* ^2^_2_	15.28	1	15.28	25.85	0.0014	significant
*X* ^2^_3_	62.25	1	62.25	105.31	<0.0001	significant
Adjust R^2^ = 0.9860						

**Table 10:** Variance analysis of rutting factor regression model.

Variance Source	Sum of Squares	Degree of Freedom	Mean Square	F Value	*p*-Value	Significance
Model	3.11	9	0.3458	53.60	<0.0001	significant
Residual error	0.0452	7	0.0065	−	−	−
Total	3.16	16	−	−	−	−
*X*_1_	1.19	1	1.19	185.02	<0.0001	significant
*X*_2_	1.25	1	1.25	193.50	<0.0001	significant
*X*_3_	0.1081	1	0.1081	16.76	0.0046	significant
*X*_1_ *X*_2_	0.0030	1	0.0030	0.4689	0.5155	not significant
*X*_1_ *X*_3_	0.0001	1	0.0001	0.0155	0.9044	not significant
*X*_2_ *X*_3_	0.0240	1	0.0240	3.72	0.0949	not significant
*X* ^2^_1_	0.0413	1	0.0413	6.40	0.0393	significant
*X* ^2^_2_	0.1744	1	0.1744	27.03	0.0013	significant
*X* ^2^_3_	0.3396	1	0.3396	52.65	0.0002	significant
Adjust R^2^ = 0.9857						

**Table 11:** Variance analysis of non-recoverable creep compliance regression model.

Variance Source	Sum of Squares	Degree of Freedom	Mean Square	F Value	*p*-Value	Significance
Model	0.1851	9	0.0206	479.83	<0.0001	significant
Residual error	0.0003	7	0.0000	−	−	−
Total	0.1854	16	−	−	−	−
*X*_1_	0.0288	1	0.0288	672.00	<0.0001	significant
*X*_2_	0.0144	1	0.0144	337.17	<0.0001	significant
*X*_3_	0.0112	1	0.0112	262.50	<0.0001	significant
*X*_1_ *X*_2_	0.0676	1	0.0676	1577.33	<0.0001	significant
*X*_1_ *X*_3_	0.0196	1	0.0196	457.33	<0.0001	significant
*X*_2_ *X*_3_	0.0144	1	0.0144	336.00	<0.0001	significant
*X* ^2^_1_	0.0152	1	0.0152	353.68	<0.0001	significant
*X* ^2^_2_	0.0026	1	0.0026	61.40	0.0001	significant
*X* ^2^_3_	0.0085	1	0.0085	198.95	<0.0001	significant
Adjust R^2^ = 0.9984						

**Table 12:** Variance analysis of creep rate regression model.

Variance Source	Sum of Squares	Degree of Freedom	Mean Square	F Value	*p* Value	Significance
Model	0.0666	9	0.0074	138.23	<0.0001	significant
Residual error	0.0004	7	0.0001	−	−	−
Total	0.0670	16	−	−	−	−
*X*_1_	0.0351	1	0.0351	655.43	<0.0001	significant
*X*_2_	0.0153	1	0.0153	285.83	<0.0001	significant
*X*_3_	0.0040	1	0.0040	75.60	<0.0001	significant
*X*_1_ *X_2_*	0.0030	1	0.0030	56.47	0.0001	significant
*X*_1_ *X*_3_	0.0001	1	0.0001	1.87	0.2141	not significant
*X*_2_ *X*_3_	0.0025	1	0.0025	46.67	0.0002	significant
*X* ^2^_1_	0.0055	1	0.0055	103.28	<0.0001	significant
*X* ^2^_2_	0.0008	1	0.0008	14.86	0.0063	significant
*X* ^2^_3_	0.0005	1	0.0005	9.95	0.0161	significant
Adjust R^2^ = 0.9944						

**Table 13:** Variance analysis of stiffness modulus regression model.

Variance Source	Sum of Squares	Degree of Freedom	Mean Square	F Value	*p* Value	Significance
Model	6060.03	9	673.34	13.60	<0.0001	significant
Residual error	346.53	7	49.50	−	−	−
Total	6406.56	16	−	−	−	−
*X*_1_	2687.91	1	2687.91	54.30	0.0002	significant
*X*_2_	1296.42	1	1296.42	26.19	0.0014	significant
*X*_3_	184.32	1	184.32	3.72	0.0950	not significant
*X*_1_ *X*_2_	604.18	1	604.18	12.20	0.0101	significant
*X*_1_ *X*_3_	21.16	1	21.16	0.4274	0.5341	not significant
*X*_2_ *X*_3_	54.02	1	54.02	1.09	0.3309	not significant
*X* ^2^_1_	16.97	1	16.97	0.3428	0.5766	not significant
*X* ^2^_2_	52.54	1	52.54	1.06	0.3372	not significant
*X* ^2^_3_	1090.39	1	1090.39	22.03	0.0022	significant
Adjust R^2^ = 0.9459						

**Table 14:** Validation test results of RSM.

Performance	Regenerant Dosage	New Asphalt Content	Shearing Temperature	Penetration	Softening Point	G*/sinδ	*m*	*S*	*J_nr_*
Unit	%	%	°C	0.1 mm	°C	kPa	―	MPa	kPa^−1^
test value	8	150	160	49.6	72.5	2.87	0.364	43.17	0.773
predicted value				52.1	73.9	2.94	0.347	44.95	0.796
Relative error (%)	―	―	―	−5.04	―	2.44	+4.67	−4.12	−2.97

## Data Availability

Not applicable.
